# Trauma Care Scenarios Following Road Traffic Crashes in Bangladesh: A Scoping Review

**DOI:** 10.9745/GHSP-D-22-00053

**Published:** 2023-04-28

**Authors:** Bushra Zarin Islam, Samiun Nazrin Bente Kamal Tune, Nahitun Naher, Syed Masud Ahmed

**Affiliations:** a Centre of Excellence for Health Systems and Universal Health Coverage, BRAC James P. Grant School of Public Health, BRAC University, Dhaka, Bangladesh.

## Abstract

**Introduction:**

We conducted a scoping review of the trauma care situation following road traffic crashes (RTCs) in Bangladesh to inform the design of a comprehensive program for mitigating associated morbidity and mortality.

**Methods:**

We used the Preferred Reporting Items for Systematic Reviews and Meta-Analysis approach to select relevant articles, documents, and reports following a set of inclusion and exclusion criteria. In total, 52 articles and 8 reports and program documents were included in the analysis. We adopted a mixed studies review method for synthesizing evidence and organized information by key themes using a data extraction matrix.

**Results:**

Findings revealed RTC mortality to be 15.3 per 100,000 population in 2019. Pedestrians, cyclists, and motorcyclists were the most vulnerable groups succumbing to moderate to grave injuries. We found that 81% of motorcycle victims did not use any safety device, an estimated 1,844 people per day suffered different degrees of injury, and 29 people per day became permanently disabled. The ambulance-based prehospital care operated in a disjointed and disorganized manner without standard operating procedures and dispatch structure. This disorganization and a lack of a universal communication system led to treatment delay, resulting in chronic disability for the victims. Injury-related patients occupied about 33% of hospital beds, 19% of which were RTC victims. The cost of care for these victims involved substantial out-of-pocket spending, which sometimes reached catastrophic levels. Since 2009, the management of RTCs has deteriorated with a concomitant increase in morbidity and mortality, resulting in a drain on people’s lives and livelihoods.

**Conclusion:**

The current situation regarding post-crash care in the country, especially when RTCs are on the rise, is not compatible with reaching the SDG targets 3.6 and 11.2 or the government’s stated goal of achieving universal health coverage by 2030.

## Abstract (precis)

For victims of road traffic crashes in Bangladesh, the prehospital care is rudimentary and the hospital-based care is often inadequate, which frequently results in catastrophic health costs for them and their families.

## Teaser

**Key Findings:**

- Bangladesh has a gradually rising high morbidity and mortality rate related to road traffic crashes (RTCs). Despite this, the availability of extensive post-crash care is nearly nonexistent.
- The prehospital care that a patient receives following an RTC is rudimentary, and hospital-based care is often inadequate. This frequently results in catastrophic health costs and consequences for the victims and their families.
- These conditions will likely obstruct the achievement of the Sustainable Development Goals by 2030.

**Key Implications:**

- To reduce RTC-related mortalities and morbidities and catastrophic health expenditure, policymakers should take measures to create a comprehensive post-crash care system.
- National stakeholders should come forward to assist the government, including by providing the required logistical assistance for effective prehospital care to intervene in the initial hours following an RTC.

## INTRODUCTION

Globally, road traffic injury is responsible for 1 death every 24 seconds and is the eighth leading cause of death.^1^ Low-income countries bear the burden of 13% of all such deaths, despite owning only 1% of all motor vehicles globally. Fifty-four percent of the fatalities occur among pedestrians, including cyclists and motorcyclists.

The most recent statistics show that Bangladesh has a high mortality rate from road traffic injury (15.3 per 100,000 population)^2^ and the highest fatality rate per 10,000 vehicles globally (102 deaths per 10,000 motor vehicles).^3^ Two-thirds of all RTC fatalities occur while transporting the patient from the accident location to a health facility, and another three-fourths do not receive any prehospital first aid at all.

The economic loss from RTCs is estimated to be about 2%–3% of the gross domestic product of low and middle-income countries,^4^ which is a challenge to development. As in other low-income countries, RTCs disproportionately affect the poor in Bangladesh,^5^ have negative consequences for the household’s economic well-being, and are a driver of poverty for many such households. This is a major system failure and hampers the Bangladesh government’s commitment to achieving universal health coverage by 2030.^1^

RTCs disproportionately affect the poor in Bangladesh and are a driver of poverty for many such households.

Thus, the issue has become a public health concern and a development challenge. To address this, in 2010, the United Nations General Assembly called for a Decade of Action for Road Safety 2011–2020 to reduce, prevent, and reverse the mortality and morbidity associated with RTCs.^6^ This goal is also reinforced by the health-related global goal for sustainable development (SDG 3.6), where the target is to halve the number of deaths and disabilities caused by RTCs by 2030. Moreover, SDG 11.2 emphasizes providing access to safe, affordable, accessible, and sustainable transport systems for all, improving road safety with special attention to vulnerable populations by 2030. The Decade of Action for Road Safety 2011–2020^3^ ended in 2020; however, Bangladesh is far from realizing its outlined objectives.^3^

Detailed information on current prehospital and hospital-based trauma care services is essential to the design and development of a professional post-crash care system in Bangladesh.^7^ Intervening in a timely manner with a prehospital care system can also decrease the post-crash morbidity and mortality rate among RTC victims. Given the urgency of the matter, we conducted a scoping review of available literature on the topic, including government reports on relevant policies and strategies, details of a “Good Samaritan” legislation, and programs by nongovernmental organizations (NGOs) and civil society organizations. As opposed to a systematic review, a scoping review provides a far broader perspective on the subject, making it a better tool to assess the matter of interest.^8^ The knowledge gained from the scoping review is expected to provide insight into the gaps, challenges, and opportunities on the topic and facilitate the development of a comprehensive program by the government.

## METHODS

We conducted a scoping review to assess the existing prehospital and hospital-based trauma care services following RTCs in Bangladesh.^9^^,^^10^ A 5-step framework adapted from the framework of Arksey and O’Malley^8^ was used for this review^11^: (1) identify the research question; (2) identify relevant studies; (3) select studies; (4) chart the data; and (5) collate, summarize, and report the results.

We developed a protocol (Table 1) that specified the study objectives, inclusion/exclusion criteria, and data sources/search engines. Our choice of the number of sources and extent of the search was limited by time. Thus, we used 3 search engines (Google Scholar, PubMed, and Scopus) for peer-reviewed publications and Google for gray literature. We also visited websites of specific organizations and institutions to search for relevant documents and reports. Finally, we also searched some relevant journal websites that to our knowledge published articles on the topic, especially within the country. The search terms were fixed, and different search terms were used to find relevant documents (Supplement 1). We followed the Preferred Reporting Items for Systematic Reviews and Meta-Analysis extension for Scoping Reviews (PRISMA-ScR) checklist approach to select articles on post-crash care, including relevant policies and practices for analysis (Supplement 2).

**TABLE 1.** Protocol for Scoping Review of Trauma Care Scenarios After RTCs in Bangladesh

Research Question	What is the current situation of existing prehospital and hospital-based trauma care services following road traffic crashes in Bangladesh?		
Objective	To assess the existing prehospital and hospital-based trauma care services in Bangladesh following road traffic crashes		
Specific research objectives	To explore the current situation of existing prehospital trauma care after RTCs in Bangladesh		
	To explore the current situation of existing hospital-based trauma care after RTCs in Bangladesh		
	To understand the extent to the current post-crash care practice in Bangladesh is compliant with WHO guidelines		
Search strategy	Inclusion criteria	Following RTC post-crash, care-related study conducted in Bangladesh	
		Full-text, peer-reviewed journal articles, blogs, reports, and other gray materials (e.g., documents on institutional websites and policy documents)	
		Language: English	
	Exclusion criteria	Countries other than Bangladesh	
	Time frame	January 1, 2009 to March 20, 2021	
Data source	Peer-reviewed journals	Google Scholar, PubMed, Scopus	
	Gray literature	Google	
	Institutional websites	Road Transport and Highways Division; Ministry of Law; TraumaLink; CriticaLink; *Nirapad Sarak Chai* ("We demand safe road"); Centre for Injury Prevention and Research, Bangladesh; Centre for Rehabilitation of the Paralysed; Accident Research Institute of BUET; other engineering universities; BRAC; Bangladesh Police; World Health Organization, etc.	
	Journal websites	South-East Asia Journal of Public Health, Bangladesh Journals Online	
Key Search Terms Used^a^			
Trauma Care (combined by “OR”) (a)		Health Sector (combined by “OR”) (b)	Geographic Location (combined by “OR”) (c)
Post-crash care after RTC, trauma care after RTC, road crash care, road traffic accident, RTC, injury from RTC, head injury from RTC, limb injury from RTC, fracture from RTC, abdominal injury from RTC, injury prevention, fatality of RTC, cost of trauma care, financing of trauma care, access to trauma care, quality of trauma care, governance of trauma care, rehabilitation after RTC, disability from RTC		Community clinics, union subcenter, upazila health complex, primary health care centers, secondary level hospitals, government district hospitals, public hospitals, private hospitals, trauma center	Bangladesh (including 64 districts)

Abbreviations: BUET, Bangladesh University of Engineering and Technology; RTC, road traffic crash; WHO, World Health Organization.

^a^Groups a, b, and c were combined with Boolean operator “AND.”

Two authors (BZI and SNBKT) searched peer-reviewed articles and reviews, commentaries, conference proceedings, and editorials published between January 1, 2009 and March 20, 2021. After removing duplicate articles, the authors independently reviewed each abstract and included articles and documents in the analysis. The authors also incorporated relevant citations from selected articles in the analysis. They critically appraised the selected articles under the guidance of the principal investigator, as per the PRISMA-ScR checklist. Because different types of evidence were reviewed, a checklist for each source was used following the JBI manual for evidence synthesis (https://jbi-global-wiki.refined.site/space/MANUAL).

### Data Extraction and Analysis

We followed the PRISMA-ScR approach to select articles on Bangladesh’s existing post-crash care scenario for analysis (Figure).

**FIGURE:**
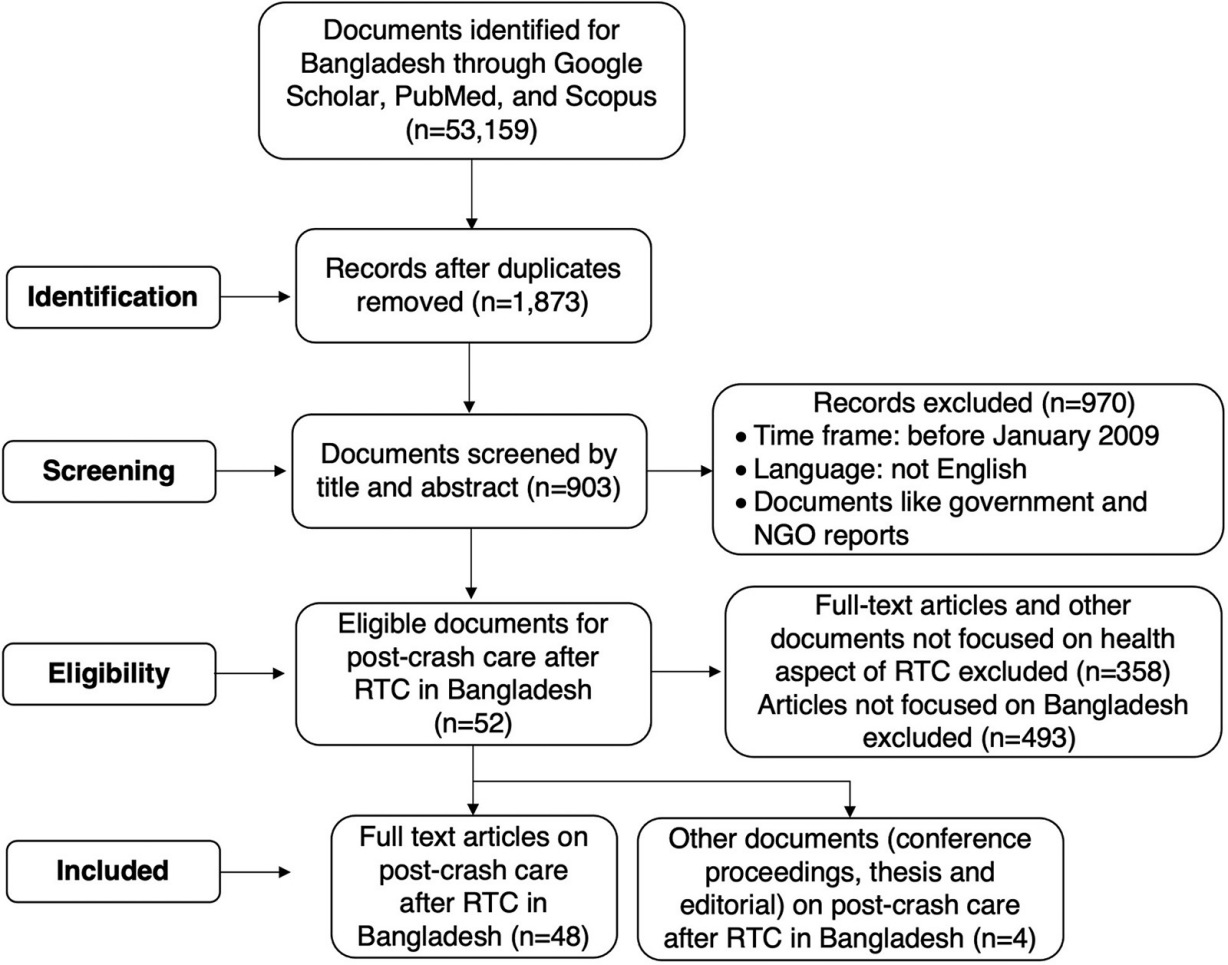
PRISMA Diagram for Selecting Documents on Care After Road Traffic Crashes in Bangladesh Abbreviations: NGO, nongovernmental organization; PRISMA, Preferred Reporting Items for Systematic Reviews and Meta-Analysis; RTC, road traffic crash.

A mixed studies review method was used for synthesizing evidence from the studies.^12^ Studies mainly focused on RTC and post-crash care scenarios in Bangladesh. Differences in the thematic (or subthematic) grouping or interpretation of the evidence in the documents were resolved by discussion among the group and with the PI to reach a consensus, with additional revisions as deemed necessary. A data extraction matrix was used for organizing data, disaggregated into key themes (with relevant subthemes) of the study (Table 2).

**TABLE 2.** Framework for Analysis of Trauma Care Scenarios Following RTCs in Bangladesh

Themes	Subthemes
Characteristics of RTCs	Magnitude, fatalities, and causes of RTCs
	Common locations
	Commonly involved vehicle
	Common pattern of injury from RTCs
	Common victims
Existing trauma care	Prehospital care (bystander and ambulance based)
	Action taken
	Access to trauma care
	Type of care received at facilities
	Follow-up on trauma care
Cost and financing of trauma care	Cost of trauma care
	Financing of trauma care
Consequences of RTCs	Patient care after RTCs
	Disability after RTCs
	Rehabilitation after RTCs

Abbreviation: RTC, road traffic crash.

Eight of 11 available reports were identified and included; 3 were discarded as these pre-dated the review period.

### Ethical Approval

Ethical approval for this study was obtained from the BRAC James P. Grant School of Public Health of BRAC University, Institutional Review Board (Protocol No. 2020-004-IR). The study involved no interaction with human subjects, and data were used for research purposes only.

## RESULTS

Search findings revealed a poor situation of post-crash care in Bangladesh. The final 52 documents in the analysis included: 1 survey finding, 38 cross-sectional studies, 4 secondary data analyses (including 1 of surveillance data), 1 document review, 1 document review with secondary data analysis, 1 non-systematic review, 2 qualitative studies, 1 conference proceedings or abstract only, 2 theses, and 1 editorial. Of these, 37 were articles on characteristics of RTCs, 9 on trauma care, 4 on cost and financing of trauma care, and 2 on consequences of RTCs. Some articles overlap in these themes. Some articles addressing the multisite issue were not included for review, as data could not be disaggregated for Bangladesh. We present the findings according to the documents retrieved and analyzed. Findings from peer-reviewed journal articles are summarized in Table 3.

**TABLE 3.** Summary of Findings From Peer-Reviewed Journal Articles on Trauma Care Scenarios Following RTCs in Bangladesh

Author (Year)	Description	Themes/Subthemes	Key Findings
Baset et al. (2017)^13^	A household census survey collected data about fatal and nonfatal RTIs.	Characteristics of RTCs	RTC mortality and morbidity found in 6.8/100,000 population and 889/100,000 population. Almost 20% of RTC victims were severely injured. Highest proportion of high injury severity: passengers (37.7%) followed by pedestrians (22.4%).
Biswas (2012)^14^	An editorial on the emerging problem of RTIs in Bangladesh.	Characteristics of RTCs	The fatality rate of road crashes in Bangladesh is very high (about 60 deaths per 10,000 vehicles per year) as compared with USA (2), United Kingdom (1.4), and New Zealand (3.3). Bangladesh has the lowest motorization level, only 2 motor vehicles for 1,000 people compared to USA (765), United Kingdom (426), and New Zealand (560).
Shafiq et al. (2017)^15^	A non-systematic review of relevant documents using Google Scholar, PubMed, and relevant legal documents.	Characteristics of RTCs	Vulnerable groups are pedestrians; the majority are males. Higher mortality found in rural areas than urban areas in both Bangladesh and Nepal.
Shohag (2009)^16^	A mixed method study to investigate and analyze the present condition of children's usage and accessibility of city streets of Dhaka.	Characteristics of RTCs	More than half (54%) of the total pedestrian fatalities are children aged 6–10 years, making them the dominant age group of pedestrian fatalities.
Ahmad et al. (2015)^17^	A cross-sectional study conducted among victims of RTC in DMCH morgue.	Characteristics of RTCs	67% of victims were male and 33% were female. Most of them were pedestrians (69%), followed by passengers (26%), and the rest were drivers. Most RTCs took place on the highway (80%), followed by a cross junction (9%). Buses caused the most fatalities (37.8%), followed by trucks (17.3%).
Hyder et al. (2009)^18^	Pilot study from multicountry global childhood unintentional injury surveillance project that was based on a sequential sample of children aged <11 years of either gender who presented to the selected ED of 5 countries, including Bangladesh.	Characteristics of RTCs	Of all unintentional injuries in children, 22% were from RTCs.
He et al. (2014)^19^	Cross-sectional observational study; standardized data were collected from children aged 0–12 years at participating EDs of 5 LMICs, including Bangladesh.	Characteristics of RTCs	Falls (50.4%) and road traffic injuries (16.4%) were the most common, affecting boys more often (64.7%).
Alonge et al. (2017)^20^	Secondary data analysis of baseline census data of Saving of Lives from Drowning project analyzing descriptive measures of fatal and nonfatal injury outcomes.	Characteristics of RTCs	RTCs were a major cause of fatal injuries (17.82%). Among those aged 25–64 years, RTCs were the major cause of fatal injury both in males and females (34% vs 24%).
Baset et al. (2012)^21^	Cross-sectional study conducted to explore the magnitude of RTC mortalities and disabilities by in-person interview from a multistaged clustered sample.	Characteristics of RTCs	The overall rate of nonfatal RTCs was 134.5/100,000, and fatality was 12.9/100,000 population. Those aged 20–39 years had the highest incidence rate of 165.7/100,000. Males had a higher rate of RTC-related morbidity, mortality, and disabilities. The most common incident involved a nonmotorized vehicle and pedestrians and occurred three times more often in rural areas than urban areas.
Roy et al. (2021)^22^	Cross-sectional study conducted by structured interview among RTC victims attending 2 hospitals in Dhaka.	Characteristics of RTCs	76% were male and 24% were female. More than 40% of patients were not taken to the hospital within first hour of injury. 22% of injuries that occurred at night were more serious. Failure to take the patient to hospital within the first hour of crash and injury occurred after midnight had a higher odds of severe injury.
Nury et al. (2012)^23^	Cross-sectional study to explore trends of RTCs in Sylhet city.	Characteristics of RTCs	Zindabazar had the most fatal and crash injuries (5.8% and 39.6%). Crash by rickshaw (>35%) was higher than other vehicles.
Ahmad et al. (2018)^24^	Cross-sectional study of 152 pedestrians selected purposively at a facility, and data were collected from them by an in-person exit interview using a semistructured questionnaire.	Characteristics of RTCs	Majority of RTCs occurred at city main roads (44%) in the daytime (41%) by buses (36%). Most were victims of hit-and-run injury (84.9%). 42% and 34.9% had upper and lower limb fractures, respectively.
Jabbar et al. (2009)^25^	Cross-sectional study to explore the risk factors related to a RTC in Bangladesh, data were collected from RTC victims of 2 medical college hospitals in Dhaka.	Characteristics of RTCs	About 31% stated that the crash happened at average speed. The majority (55%) of drivers drove on the wrong side of the road. Only 6.75% of victims used protective measures during crashes.
Chowdhury et al.(2012)^26^	Cross-sectional prospective study was done in Faridpur Medical College Hospital during January–June 2011 to study victims of crashes caused by flatbed tricycles.	Characteristics of RTCs	12% of admitted patients were of RTCs involving flatbed tricycles. The most common (55.36%) patients were victims of tricycle crashes, while the maximum number of crashes (82.14%) occurred in urban areas and on highways. They are run in violation of the Bangladesh Motor Vehicles Act (1983).
Talab et al. (2018)^27^	Cross-sectional study based on Bangladesh Health and Injury Survey 2016, conducted March–June 2016 to explore the epidemiological burden of motorcycle injuries.	Characteristics of RTCs	Motorcycle injuries comprise 21.6% of all transport injuries. An estimated 1,844 people per day suffer different degrees of injury, and 29 people per day become permanently disabled due to motorcycle injuries. 81.3% of injured victims did not use any safety device.
Islam et al. (2018)^28^	Cross-sectional study; primary data were collected from 200 RTC victims from Khulna Medical College Hospital and Sathkhira Sadar Hospital about their knowledge of traffic rules.	Characteristics of RTCs	47.4% of victims reported a motorcycle as their RTC vehicle. Those who received proper treatment were almost 5 (OR 4.69) times more likely to know traffic rules than those who had not received appropriate treatment.
Anisuzzaman et al. (2019)^29^	Cross-sectional study that reviewed 437 cases of chest trauma from January 2006 to December 2015.	Characteristics of RTCs; common pattern of injury from RTC	The leading cause of chest trauma was RTC (61%).
Hakim et al. (2018)^30^	Cross-sectional study conducted on 60 patients with hepatic injury at DMCH, January 2013–December 2014.	Characteristics of RTCs; common pattern of injury from RTC	RTC was the most common liver injury mechanism in 71% of cases.
Sultana et al. (2018)^31^	Cross-sectional study undertaken to determine the epidemiological and clinical profile of patients presented with mandible fracture.	Characteristics of RTCs; common pattern of injury from RTC	RTC was the leading cause of mandible fracture (54%).
Faruquzzaman et al. (2012)^32^	Cross-sectional study was conducted in general surgery wards of Chittagong Medical College Hospital, Bangladesh.	Characteristics of RTCs; common pattern of injury from RTC	In the surgical ward, the majority (24.5 %) of the trauma patients were from RTCs.
Rahman et al. (2013)^33^	Cross-sectional study was done in the Department of Surgery at MMCH among 100 patients with traumatic gut injury.	Characteristics of RTCs; common pattern of injury from RTC	Of 100 patients with abdominal trauma, RTC was the main (58.14%) mechanism of nonpenetrating abdominal trauma.
Ara et al. (2013)^34^	Cross-sectional study in which ultrasonography was done in the Centre for Nuclear Medicine and Ultrasound at MMCH to see any intraperitoneal collection, pleural collection, or vital organ injury.	Characteristics of RTCs; common pattern of injury from RTC	The common cause of trauma was RTC (46%). Ultrasonography detected 52% of patients with intraperitoneal collection, 26% with intraabdominal organ injury, and 10% with a pleural group. The liver was the most affected organ (30%).
Islam et al. (2018)^35^	Cross-sectional study conducted in Combined Military Hospital Dhaka to explore and evaluate patterns of head injury in victims reporting to emergency and casualty.	Characteristics of RTCs; common pattern of injury from RTC	The majority (50.1%) of head injury patients were RTC victims. 53% of male head injury patients and 44.8% of female head injury patients were RTC victims.
Rahman et al. (2018)^36^	Cross-sectional study; data were collected from medical records of all spinal cord injury patients, January 2012–December 2014, at CRP, Savar.	Characteristics of RTCs; common pattern of injury from RTC	RTC was the second most common (24.7%) cause of spinal cord injury, followed by fall from height (45.8%).
Haque (2014)^37^	Cross-sectional study of 94 samples selected purposively from in-patients at CRP.	Characteristics of RTCs; common pattern of injury from RTC	RTC was the cause of spinal cord injury in 36.8% of cases.
Razzak et al. (2017)^38^	Cross-sectional study conducted in 5 hospitals on 600 cases to overview demographic characteristics of spinal cord injury in Bangladesh.	Characteristics of RTCs; common pattern of injury from RTC	RTC was the cause of spinal cord injury in 25.5% of cases.
Ahsan et al. (2019)^39^	Cross-sectional study assessing demographics, incidence, mode of trauma, associated spine injuries, complications, neurological improvement, and mortality of the multiple injury patients.	Characteristics of RTCs; common pattern of injury from RTC	In a tertiary hospital, RTC was etiology of 80% of spinal injury patients with multiple injuries.
Hossain et al. (2020)^40^	Cross-sectional study of patients admitted with pelvic fractures at 2 tertiary care hospitals in Dhaka.	Characteristics of RTCs; common pattern of injury from RTC	RTCs were the most common underlying cause of injuries among patients with pelvic fractures.
Mahmud et al. (2015)^41^	Cross-sectional study conducted in the Department of Otolaryngology and head-neck surgery of Bangabandhu Sheikh Mujib Medical University and DMCH for 6 months to see the common indication of tracheostomy.	Characteristics of RTCs; common pattern of injury from RTC	Head injury with a history of RTC was the most common (27.5%) indication of tracheostomy in the ICU. RTC with cervical spine injury was another indication of tracheostomy (5%).
Islam et al. (2011)^42^	Cross-sectional study evaluating factors influencing the outcome in patients with extradural hematoma undergoing surgery treated in a tertiary hospital in Bangladesh.	Characteristics of RTCs; common pattern of injury from RTC	RTC is the most common cause (65%) of extradural hematoma.
Chowdhury et al. (2014)^43^	Cross-sectional study of 138 patients with extradural hematoma who underwent surgery.	Characteristics of RTCs	RTC was the cause of extradural hematoma in children in 31.9% of cases. Most of the RTCs involved three-wheeled vehicles.
Ahmad et al. (2010)^44^	Cross-sectional study conducted at DMCH morgue among 100 post-mortem cases of RTC victims over 1 year.	Characteristics of RTCs, common victims, common pattern of injury from RTC	The highest number (68%) of victims were pedestrians. Apart from bruise and laceration in almost all the victims, 77% had a brain injury and 77% had injury to vital abdominal organs. Most victims had subdural hemorrhage (43%) followed by subarachnoid hemorrhage (36%).
LeBrun et al. (2013)^45^	Cross-sectional study to understand the role of surgeons and anesthetists to prevent death.	Characteristics of RTCs; common pattern of injury from RTC	The second common cause of surgical death was extensive trauma from RTC, after hemorrhage during cesarean delivery.
Rima (2016)^46^	Cross-sectional study conducted on purposively selected 100 subjects from several hospitals to determine the characteristics of amputees in Bangladesh.	Characteristics of RTCs; common pattern of injury from RTC	RTC was the cause of 60% of lower limb amputations and 85% of upper limb amputations.
Imam et al. (2020)^47^	Cross-sectional study to describe the cause, type, and consequences of lower limb amputation, cross-sectional data were collected from lower limb amputees who attended CRP.	Characteristics of RTCs; common pattern of injury from RTC	RTC was the leading cause (58.7%) of lower limb amputation. RTC was the primary cause of lower limb amputation across all age groups. RTC was the primary cause of lower limb amputation in rural (57.7%) and urban settings (60.7%).
Chowdhury et al. (2016)^48^	Cross-sectional study; data were collected from Anwara Upazila, Chittagong, by structured questionnaire from 6,265 individuals to evaluate injury epidemiology.	Characteristics of RTCs; common pattern of injury from RTC	RTC was the mechanism of injury in 30% of cases in the rural population.
Islam et al. (2018)^49^	Cross-sectional study evaluating reliability of the medial hemi-soleus muscle flap for wound coverage of infected open fracture of the distal third of the tibia.	Characteristics of RTCs; common pattern of injury from RTC	Of patients receiving hemi-soleus muscle flap for wound coverage of infected open fracture of the distal third of the tibia, 80% were RTC victims.
Talukder et al. (2013)^50^	Qualitative study using in-depth interviews and case studies to explore health care–seeking decisions and the culture of handling RTC victims from their experiences.	Existing trauma care; prehospital care, consequences of RTCs	Mostly the health care–seeking decision came from bystanders of RTCs. In most cases, RTC victims did not receive any early treatment. Instead of public hospitals, victims are often shifted to a private commercial clinic. Distance, shortage of money, lack of opportunity to get help, and lack of mass awareness were identified as primary obstacles to getting even first aid. Hence, disability is often inevitable.
Kabir et al. (2009)^51^	Document review to understand the RTC scenario of the Bonpara-Gazipur highway.	Existing trauma care; prehospital care	Victims remain untouched for hours. Some highways lack proximity to any health care provider. There is a lack of effective means of communication with health care centers. Many victims become disabled due to a lack of proper treatment and slow response time.
Maghfiroh et al. (2018)^52^	Cross-sectional observational study conducted to improve emergency medical service in Dhaka by reducing ambulance service response time.	Existing trauma care; prehospital care	Ambulances need less time to make trips compared to other modes of transport. In Dhaka city, 81.3% of patients come to the hospital by any other mode of transport than an ambulance. Repositioning ambulances in a strategic location for maximum coverage substantially improves response time.
Kamruzzaman et al. (2019)^53^	Cross-sectional study of 17,435 patients; data were collected from hospital records attending in the Casualty Department, MMCH, November 2017–May 2018, and were categorized based on mode of injury.	Existing trauma care	In a tertiary level hospital, the maximum RTCs attended in one day was 65 and the minimum was 3. Non-RTC maximum was 83 and minimum was 25.
Rahman et al. (2012)^54^	Document review and secondary data analysis of 13,305 hospital admission records to evaluate the hospital bed occupancy rate.	Existing trauma care	Among all admissions to a tertiary level hospital, 27.2% were RTC cases. RTC victims occupied 21.7% of beds.
Sultana et al. (2015)^55^	Secondary data analysis to depict the medical condition pattern of the Bangladeshi population seeking services from the district hospital.	Existing trauma care	RTC is ranked within the top 5 conditions for seeking medical care from the district hospital.
Mashreky et al. (2010)^56^	Cross-sectional study to estimate the burden of RTC patients and length of stay at the hospital, the discharge records from a few primary and secondary level hospitals were reviewed. Direct interview with the patient was also adopted to measure the cost of RTC.	Existing trauma care, cost and financing of trauma care	Injury patients occupied 33% of primary and secondary hospitals with >19% of them RTC victims. 39% of all RTC patients were hospitalized and cured. The average length of hospital stay was 5.7 days. The average cost for each RTC patient was US$86 (BDT 5834).
Ahmed et al. (2010)^57^	Cross-sectional study conducted to understand the primary cause of hospitalization in rural Bangladesh.	Existing trauma care	17.7% of hospitalizations were due to injuries. 9% of those injuries were from RTCs. Those aged 15–45 years were the most common age group hospitalized due to RTC (3.7%).
Zaman (2013)^58^	Ethnography to show how social dynamics are necessary to deal with the structural realities and limitations of the hospital by participant observation.	Existing trauma care	A subject of this ethnography is an RTC victim whose wife remains by his side at the hospital for proper care. Rather than doing nursing, Bangladeshi nurses are mostly occupied with administrative work. Most of the caregivers were male family members.
Karim et al. (2011)^59^	Cross-sectional study conducted on 55 admitted RTC cases to determine the economic impact of RTC on the patients.	Cost and financing of trauma care, consequences of RTCs	The victims were mainly male (85%). 27.3% had mild, 54.5% moderate, and 18.2 % severe injuries. The maximum length of hospital stay was 15–40 days for 40% of cases. Average direct cost was BDT5455,^a^ average indirect cost was BDT8440. The total average cost of injury was BDT13277. 45% of victims became disabled.
Dalal et al. (2009)^60^	Cross-sectional study conducted to study the nature and extent of out-of-pocket expenses for unintentional injuries using rural Bangladesh.	Cost and financing of trauma care	Road traffic injuries comprised 10% of out-of-pocket payments of victims. Instead of relying on public health care facilities, most rural Bangladeshi spend their own money on treatments.
Alam et al. (2016)^61^	Secondary data analysis to assess the economic consequence of RTIs on households in South Asia (including Bangladesh).	Cost and financing of trauma care	RTI-affected households had significantly higher levels of out-of-pocket health spending. Indicators of catastrophic spending were also significantly higher in RTI-affected households.
Alam (2018)^62^	Qualitative thesis to learn the livelihood patterns of road crash survivors, focus group discussions were conducted with 19 subjects, and some document review.	Cost and financing of trauma care, consequences of RTCs	Prolonged treatment of RTC survivors often becomes a catastrophic health expenditure. Disability itself was the main factor in the vulnerability of RTC victims. A few victims were fully dependent on their family members.
Razzak (2013)^63^	Cross-sectional observational study to explore the outcome of current practices in treatment of 56 patients with traumatic spinal cord injuries in Bangladesh.	Consequences of RTCs	After comprehensive rehabilitation over an average of 114 days, 28.6% had achieved neurological improvement. At discharge, 20.8% did not need any assistive device.
Sarkar et al. (2018)^64^	Cross-sectional study to observe the actual scenario of developing physiotherapy services as part of primary health care services at district hospitals in Barisal division.	Consequences of RTCs	Of patients seeking physiotherapy, 9.1% were victims of RTCs.

Abbreviations: BDT, Bangladeshi taka; CRP, Centre for Rehabilitation of the Paralyzed; DMCH, Dhaka Medical College Hospital; ED, emergency department; ICU, intensive care unit; LMIC, low- and middle-income country; MMCH, Mymensingh Medical College Hospital; OR, odds ratio; RTC, road traffic crash; RTI, road traffic injury.

^a^105 Bangladeshi taka=US$1.

### Peer-Reviewed Journal Articles

#### Characteristics of RTCs

In rural Bangladesh, mortality from RTCs was found to be 6.8 per 100,000 population per year and morbidity to be 889 per 100,000 population per 6 months from a household survey in 2013.^13^ However, the national mortality rate due to RTC was 15.9 per 100,000 population in the same year.^2^ According to the Accident Research Centre at the Bangladesh University of Engineering and Technology, the country has a very high RTC fatality rate of about 60 deaths per 10,000 vehicles per year and the lowest motorization level—only 2 motor vehicles for 1,000 people^14^— compared to any other country. Pedestrians of different age groups were found to be most vulnerable to RTCs.^15^^–^^17^ It was a leading cause of unintentional injuries in children^18^^,^^19^ and a leading cause of fatal injury among adults aged 25–64 years for both males and females.^20^ Males were found more vulnerable than females.^18^^,^^20^^–^^22^ Buses were found to cause the most crashes, while rickshaws were in frequent accidents on urban roads.^17^^,^^23^^,^^24^ Of RTC victims, 31% stated that crashes were happening even when the vehicles were driving at an average speed because most drivers (55%) drove on the wrong side of the road.^25^ At the district level, a peripheral tertiary hospital observed that 12% of the RTC victims suffered injuries from makeshift 3-wheelers, locally known as *nasimon* and *karimon*, which have been illegally riding on district highways.^26^ In recent years, with the increase in motorcycles on the road, crashes involving these vehicles have also become more frequent.^27^^,^^28^ This is a matter of growing concern as 81% of injured victims of motorcycle crashes did not use any safety device; thus, an estimated 1,844 people per day suffered from different degrees of injury and 29 people per day became permanently disabled due to injuries caused by motorcycle crashes.^27^

Bangladesh has a very high RTC fatality rate of about 60 deaths per 10,000 vehicles per year and the lowest motorization level compared to any other country.

RTCs result in a cascade of problems beginning with physical injuries, including limb fractures, blunt trauma to the liver and/or chest, and damage to vital organs.^24^^,^^29^^–^^31^ In the surgical ward of a large tertiary hospital, RTC was found to be a significant reason for patient admissions (24.5%).^32^ RTC was the most common mode (58%) of nonpenetrating abdominal trauma^33^ and gut injury.^34^ Severe injuries, like head or spinal cord injury, also crippled a substantial number of RTC victims.^35^^–^^38^ In a tertiary hospital, it was found that RTC was responsible for 80% of spinal injury patients with multiple injuries.^39^ Among the patients with a pelvic fracture at 2 tertiary hospitals in Dhaka, RTC was the major cause.^40^ RTC victims with a head injury and spinal cord injury often undergo lifesaving procedures like tracheostomy for survival.^41^ RTC was the most common cause (65%) of extradural hematoma.^42^ Even pediatric victims of RTCs often suffered from extradural hematoma.^43^ Postmortem examination of RTC victims revealed a substantial proportion with subdural (43%) and subarachnoid hematoma (36%).^44^ Extensive trauma from RTCs was the second most common cause of surgical death.^45^ However, even upon survival, a large proportion of RTC victims required an amputation.^46^^,^^47^ RTC was the major cause of lower limb amputation in a rural setting (57.7%)^47^ and 30% of injuries in the rural population.^48^ Even if they survive, RTC victims with a wound, such as an open tibial fracture, sometimes must undergo a highly invasive procedure.^49^

#### Prehospital Trauma Care

After an RTC, on-the-spot emergency care entirely depended on the bystanders and their knowledge and awareness, the location of a nearby health care facility, and the facility’s capacity to manage RTC victims properly.^50^ Often, the victims received no early treatment, let alone first aid (which the victims received after 2–12 hours). The most recent evidence also shows that more than 40% of RTC victims were not taken to the hospital within the first hour of injury, which often contributed to higher odds of severe injury.^22^ Limited availability of ambulances/transportation, weak emergency communication systems, distance to health facilities, victims’ financial condition, and lack of skills for rapid management of RTC victims were identified as factors that hindered proper prehospital and hospital care.^50^^,^^51^ In Dhaka, 81% of patients came to the hospital by transport other than an ambulance (e.g., private vehicles), as they have a faster response time.^52^ Three-wheeled scooters and rickshaws were also used for shorter trips. However, after comparing ambulance logs and emergency room admission logs, it was found that ambulances spent less time making the same trip than other transport modes. Evidence showed that repositioning ambulances either close to accident-prone points or near strategically important health care facilities substantially improved response time in 90% of cases.^52^

Evidence showed that repositioning ambulances either close to accident-prone points or near important health care facilities improved response time in 90% of cases.

#### Facility-Based Trauma Care

Victims were often transported by bystanders at the accident location to the (for-profit) private hospital rather than the government hospital.^52^ The different levels of hospitals in Bangladesh were found to be occupied with RTC victims at varying degrees. A tertiary-level hospital attended from 3 to 65 RTC patients in a day.^53^ Among all tertiary-level hospitals, 27% of cases attended were found to be from RTCs, while 22% of the beds were occupied by RTC victims.^54^ In secondary-level hospitals, RTC was 1 of the top 5 reasons for seeking emergency health care at the facility.^55^ Approximately 39% of RTC victims needed hospitalization from those attending emergency care, 10% were referred to a higher center, and about 51% were managed by first aid. In another study, about 33% of beds of primary and secondary hospitals were occupied by injury-related patients, 19% of which were RTC victims.^56^

The majority of RTC victims were aged 18–45 years, accounting for 70% of total RTC-related admissions in these facilities, and more than two-thirds of them were male.^56^ At discharge, 39% of the hospitalized RTC victims were completely cured, 30% left with some improvements, 12% were referred to higher-level hospitals, and another 18% decided to leave the hospital without completing treatment.^56^ In upazila health complexes, the injury was the reason for 18% of hospitalizations and 9% of those injuries were from RTCs, mostly among individuals aged 15–45 years.^57^ In a hospital ethnography, it was observed that, with a few exceptions, the majority of the patients’ caregivers (including the RTC victims) were male, dubbed “silent saviors.”^58^ They provided emotional support to patients as hospital staff seldom had time to converse with them beyond what was relevant to treatment.

#### Cost and Financing of Trauma Care

Findings reveal the average length of hospital stay to be 5.7 days and cost per RTC patient to be 5834 Bangladeshi taka (US$56), not considering the victim’s inability to earn an income during hospitalization or the opportunity costs of attendants or caregivers. It also did not include the cost of hospital services, consultation fees, and medication provided by the facilities (as they were public hospitals). It represented only the amount spent by patients for food, travel, accommodation (including for the attendants), and medicine, which was not available in hospitals.^56^ A different scenario emerged when direct and indirect costs were calculated in a study from the National Institute of Traumatology and Orthopedic Rehabilitation in Dhaka, which amounted to a total average cost for RTCs of 13277 Bangladeshi taka (US$126).^59^

Only 14% of victims received government medical assistance, while 17% benefited from public health care and 13% from private health care; the remainder of the cost was covered by out-of-pocket (OOP) expenditure by the patient and their family.^60^ Thus, instead of relying on public health care facilities, most rural Bangladeshi people spent money from their own pockets for treatment.^60^ Based on the World Health Survey 2012, a secondary data analysis determining OOP costs for South Asian households (including Bangladesh) shows households affected by road traffic injury to have significantly higher levels of OOP health expenditure per member (US$0.75, *P*<.01), higher OOP expenditure on drugs per member (US$0.30, *P*=.03), and higher OOP hospital expenditure per member (US$0.29, *P*<.01) in the 4 weeks preceding the survey.^61^ The qualitative finding also showed that due to prolonged treatment costs, the RTC victim and/or family developed poverty after the crash.^62^

Instead of relying on public health care facilities for post-crash care, most rural Bangladeshi people spent their own money for treatment.

#### Consequences of RTCs

Lack of appropriate transportation (e.g., ambulance equipped with resuscitation instruments) and a rapid communication system led to treatment delay, resulting in chronic disability for the victims for the rest of their lives.^50^ Over 45% of cases became disabled, 25% turned handicapped, and 22% suffered from mental stress and shock.^59^ Because disability limits a survivor’s capacity to earn an income, it compromises their economic conditions; a few survivors became fully dependent on their family members.^62^ Some of the victims were able to receive rehabilitation treatment. For example, among a cohort of spinal cord injury patients, within an average of 114 days, 29% achieved a neurological improvement of various grades, 21% did not need any assistive device, and 49% required wheelchairs.^63^ Even at a district hospital, among patients who came to seek physiotherapy, 9.1% were found to be victims of RTCs.^64^

### Gray Literature

Eleven relevant reports on post-crash care were identified, of which 8 have been reviewed; the remaining 3 pre-date the review timeline. In the past decade, quite a number of studies on this topic have been commissioned by various stakeholders and research organizations. A World Health Organization South-East Asia Regional Office report in 2009 painted a dismal picture of road safety conditions in the region. It noted the presence of at least 1 law related to the major risk factors (e.g., speeding, drunk driving, helmets, seatbelts, and child restraints) in each country but with poor implementation.^65^ The most informative study was done in 2013 by 2 major organizations, BRAC and the Power and Participation Research Center, which considered road safety issues and tried to identify action points. The study emphasized updating the list of accident-prone points for action planning and improvement, establishment of quality driving schools and traffic training institutes, and the urgency of instituting first responder care, including a national emergency call number.^66^ Another report of a nationwide cross-sectional survey of households in Bangladesh mentioned the training of first responders on first aid and protecting the “Good Samaritans,” as well as additional investments for achieving the SDG goal.^67^ Other suggested measures include improving the safety of buses and trucks, which are the major vehicles striking pedestrians^68^; 9 priority sectors for action for road safety^69^; and better engineering of road safety aspects during construction of roads and highways.^70^ Reviews revealed that the condition of road safety deteriorated in Bangladesh compared to its neighbors over time, and a new vision is required to change the very discouraging situation.^1^^,^^5^ These findings from reports and documents are summarized in Supplement 3.

Reviews revealed that the condition of road safety deteriorated in Bangladesh compared to its neighbors over time.

### Activities of NGOs, Voluntary Organizations, and Civil Society Organizations

A number of NGOs, voluntary organizations, and civil society organizations have come forward to fill in the gaps and advance the cause of road safety in Bangladesh, including through attempts to develop different models of post-crash care. Most efforts are small, but further scaling up these initiatives may be a game-changer in this sector (Supplement 4).

The most vocal and active among these initiatives is the *Nirapad Sarak Chai* (“We demand safe road”) movement, which developed a volunteer-based first responder cadre from the local community.^71^ Its programs also include training for new drivers and transport laborers, refresher training for existing drivers and transport laborers, and school-based awareness raising. Other organizations working with a similar idea of developing first responders from the local community include TraumaLink (covering the Dhaka-Chittagong, Dhaka-Mymensingh, and Dhaka-Aricha highways)^72^ and CriticaLink (working mainly in and around Dhaka).^73^ These organizations use a mobile phone and apps-based communication system to mobilize their volunteers whenever necessary within their areas of operation. The Centre for the Rehabilitation of the Paralysed is involved with comprehensive rehabilitative care of trauma victims,^74^ while the Centre for Injury Prevention and Research, Bangladesh conducts research on the injuries for evidence-based interventions.^75^ BRAC, an indigenous NGO with overseas operations, has a comprehensive road safety program comprising a driving school to train drivers, a multisectoral program to engage stakeholders to tackle the root causes of RTCs, and an advocacy component for policymakers and communities to take action.^76^ The Accident Research Institute is a dedicated accident research organization in the country and conducts accident investigation and reconstruction, road safety audits, and training on road safety for professionals.^77^ Lastly, the demand-side *Jatri Kalyan Samiti* (Passenger Welfare Association) keeps monthly records of RTCs and details of victims and advocates for road safety from the perspective of road users.^78^

## DISCUSSION

We conducted this scoping review to understand the current situation related to existing post-crash care services following RTCs in Bangladesh. We adopted the PRISMA-ScR approach to retrieve relevant published and gray literature. A mixed-studies review method was used to organize the extracted materials from the searched documents and analyze them by key study themes.^12^ Findings reveal important insights into the poor management of RTCs, including their rising numbers and multidimensional effects on health and well-being and lives and livelihood. More importantly, we found poor post-crash care services in the country, almost nonexistent first-responder and professional ambulance care, and a high cost of post-crash hospital care and rehabilitative care. We discuss the implications of these findings for the informed design of a comprehensive post-crash care system following the pillars of the United Nations Global Plan for Road Safety.^6^

Findings reveal important insights into the poor management of RTCs, including their rising numbers and multidimensional effects on health and well-being.

### Road Safety Management

It is disheartening to note from the reviewed literature that the road safety performance of Bangladesh is not just poor but has been deteriorating since 2009. This is also corroborated by reports from *Jatri Kalyan Samiti*.^79^^,^^80^ According to its most recent report, deaths from RTCs increased by 8% in 2019 compared to 2018,^80^ not unlike other low- and middle-income countries in Asia.^81^^,^^82^ Poor traffic management—including manual traffic control, lack of bus stops leading to passengers waiting in the road, and halfhearted implementation of traffic laws—is a common phenomenon in the streets of large cities like Dhaka. Combined with this are the outdated vehicles on the road driven by poorly trained drivers, poor awareness, and unwillingness to follow traffic rules. The traffic and road safety rules created by the people for their convenience have resulted in a horrific situation.^83^ All of this deterioration occurred during a time period that fell within the United Nations Decade of Action for Road Safety.^1^

### Safer Roads and Road Users

Findings revealed vulnerable road users—including pedestrians, cyclists, and, more recently, motorcyclists—to be the most frequent victims of RTCs in Bangladesh, as is also common in other Southeast Asian countries.^84^^–^^86^ This is not unexpected; the lack of population awareness, occupation of footpaths by street vendors, and absence of dedicated routes for cyclists increase the risk for involvement of vulnerable road users in RTCs. The traffic systems in large cities need to be redesigned to address these problems (e.g., dedicated footpaths for pedestrians only; dedicated routes for bicycles, motorcycles, and 3-wheelers; and discouragement of slow-moving and fast-moving vehicles occupying the same lane).

One of the major problems on the highways is the variation in traffic speeds, as observed in the findings. In addition, faulty road construction with sharp bends, narrow or absent shoulders, and marketplaces next to highways have contributed to more severe highway crashes. Thus, the highways also need to be designed for fast-moving traffic.

### Post-Crash Response

The importance of first aid for RTC victims’ survival cannot be overemphasized, and yet first aid response is almost nonexistent in Bangladesh, except for some work undertaken by NGOs on a very small scale. This contrasts with other countries in the region, such as India,^87^ Nepal,^88^ Sri Lanka,^89^ and Thailand,^90^ which all have well-developed post-crash care systems on the ground, including first-responder care. Access to post-crash care (i.e., availability of an ambulance or other transport for the transfer of trauma victims) is also problematic. Some private for-profit organizations provide this service in makeshift vehicles, but this cannot always guarantee professional attendance and needed resuscitation equipment. From the review findings, it is obvious that first-responder care needs to be organized in an effective way with help from public, private, and NGO sectors, based on experience from neighboring countries in the region. The small initiatives by NGOs such as TraumaLink^72^ can be evaluated for effectiveness and efficiency, and the successful ones could be used as a model for scaling up nationally.

Review findings show that first-responder care needs to be organized in an effective way with help from public, private, and NGO sectors.

### Cost of Post-Crash Care

Road safety has become a development issue due to its effects on gross domestic product, especially for low- and middle-income countries like Bangladesh.^91^ The significant number of RTCs and consequent costs of post-crash and rehabilitative care have a negative effect on human and economic development. As revealed in our findings, the cost of immediate and long-term rehabilitative care for trauma victims involves substantial spending, primarily through out-of-pocket expenditure by victims and their families. Sometimes this may reach the level of catastrophic expenditure (e.g., as shown in India^92^ and other South Asian countries^61^). Since the ability to earn income is affected, many households in Bangladesh succumb to deteriorating economic conditions, ultimately sliding into poverty.^60^ Thus, providing necessary and prolonged care free of cost or at subsidized cost will go a long way to revive the income-earning potential of the victims and their families and contribute to the poverty alleviation strategy of the government. The public secondary facilities at the district hospitals adjacent to highways need comprehensive hospital-based trauma care to reduce morbidity and mortality and out-of-pocket expenditure.

### Rehabilitative Care

Finally, an increase in skills and innovations in rehabilitative care is needed. Since its independence, Bangladesh has already gained some infrastructure for specialized rehabilitative care in the public and private sectors. This care began with serving the needs of the wounded freedom fighters. However, current capacity is not commensurate with the increasing demand in the public sector and is costly in the for-profit private sector. As revealed in the review, some NGOs and nonprofit organizations provide specialized and skilled services. These can be integrated into the comprehensive post-crash care system and resources allocated by the government to scale their services as a stopgap measure.

### Limitations

A more extensive search could not be done due to constraints in time and resources. In addition, there was a dearth of published materials in Bangladesh, as it is a less-researched country. However, given the number and types of documents (both peer-reviewed and gray literature) found, we are confident about reflecting the actual scenario in its different dimensions and entirety.

Because this is a scoping review of published and unpublished documents and not a study of the actual scenario, we recommend conducting a survey on the relevant issues on a representative sample backed up by in-depth qualitative exploration and identifying action points that align with global recommendations. In addition, the issue of catastrophic costs of the RTCs, including financing of the relevant reforms, warrants further analysis to inform the policymakers and program implementers for designing appropriate interventions.

## CONCLUSIONS

Over the years, the management of RTCs in Bangladesh has deteriorated with a concomitant increase in morbidity and mortality, placing a strain on people’s lives and livelihoods. Due to its effects on household income earning, this has become both a development problem and a public health problem. The near absence of prehospital care and inadequate hospital-based care for RTC victims is tragic and unacceptable. It needs to be addressed urgently to reach the government’s stated goal of achieving universal health coverage by 2030 and the SDG targets of 3.6 and 11.2.

To develop effective and efficient post-crash care in the country according to World Health Organization recommendations, the government should earmark a substantial proportion of the annual health budget throughout the current decade. In addition, the technical aspects of “safe roads, safe vehicles, and safe drivers” should be given proper attention for remedial measures. It is encouraging to note that, after many delays, the government finally passed the much-anticipated Road Transport Act 2018^93^ in October 2018,^94^ which emphasizes treating and compensating RTC victims.

## Supplementary Material

22-00053-Islam-Supplements.pdf

## Notes

**Peer Reviewed**

**First published online:** April 17, 2023.
