# Supplementary material for: Trauma Care Scenarios Following Road Traffic Crashes in Bangladesh: A Scoping Review
Source: Glob Health Sci Pract. 2023 Apr 28;11(2):e2200053. doi: 10.9745/GHSP-D-22-00053 (PMC10141435; doi:10.9745/GHSP-D-22-00053)
Supplement: 22-00053-Islam-Supplements.pdf [file 22-00053-Islam-Supplements.pdf]

Supplement to: Islam BZ, Tune SN, Naher N, Ahmed SM. Trauma care scenarios following road traffic crashes in Bangladesh: a scoping review. *Glob Health Sci Pract*. 2023;11(2):e2200053. https://doi.org/10.9745/GHSP-D-22-00053

Supplement 1. Full electronic database search strategy for PubMed

With timeframe (1st January 2009- 20th March 2021. Following combination of search terms were used:

Database	PubMed
Strategy no.	Combination search terms
1.	(((post-crash care after RTA) OR (trauma care after RTA) OR (road crash care) OR (Road Traffic Accident) OR (RTA) OR (injury) OR (head injury from RTA) OR (limb injury from RTA) OR (fracture from RTA) OR (abdominal injury from RTA) OR (injury prevention) OR (fatality) OR (cost of trauma care) OR (financing of trauma care) OR (access to trauma care) OR (quality of trauma care) OR (governance of trauma care) OR (rehabilitation after RTA) OR (disability from RTA)) AND ((community clinics) OR (union sub centre) OR (upazila health complex) OR (primary health care centers) OR (secondary level hospitals) OR (government district hospital) OR (hospitals) OR (public hospitals) OR (private hospitals) OR (trauma center)) AND (Bangladesh)))
2.	((trauma care after RTA) AND (Hospitals) AND (Bangladesh))
3.	((Road Traffic Accident) AND (Public Hospitals) AND (Bangladesh))
4.	(((injury from RTA) OR (fatality of RTA)) AND ((Government district hospitals) OR(Public Hospitals) OR (Secondary level hospitals)) AND (Bangladesh)))
5.	(((post crash care after RTA) OR (road crash care)) AND ((primary healthcare centers) OR(Public Hospitals) OR (upazila health complex)) AND (Bangladesh)))
6.	(((rehabilitation after RTA) OR (disability from RTA)) AND ((private hospitals) OR(Public Hospitals) OR (trauma center)) AND (Bangladesh)))
7.	(((financing of trauma care) OR (cost of trauma care)) AND ((private hospitals) OR(Public Hospitals)) AND (Bangladesh)))
8.	(((access to trauma care after road crash) OR (quality of trauma care after road crash) OR (governance of trauma care after road crash)) AND (hospitals) AND (Bangladesh)))
9.	(((financing of trauma care) OR (cost of trauma care)) AND ((Government district hospitals) OR (Public Hospitals) OR (Secondary level hospitals) OR (primary healthcare centers)) AND (Bangladesh)))
10.	(((access to trauma care after road crash) OR (quality of trauma care after road crash) OR (governance of trauma care after road crash)) AND ((Community clinics) OR (Union sub-center)) AND (Bangladesh)))

Supplement 2. Preferred Reporting Items for Systematic reviews and Meta-Analyses extension for Scoping Reviews (PRISMA-ScR) Checklist for "Trauma care scenario following Road Traffic Crashes (RTCs) in Bangladesh: A Scoping Review"

SECTION	ITEM	PRISMA-ScR CHECKLIST ITEM	REPORTED ON PAGE #
**TITLE**			
Title	1	Identify the report as a scoping review.	Yes, P-1
**ABSTRACT**			
Structured summary	2	Provide a structured summary that includes (as applicable): background, objectives, eligibility criteria, sources of evidence, charting methods, results, and conclusions that relate to the review questions and objectives.	Yes, P-1-2
**INTRODUCTION**			
Rationale	3	Describe the rationale for the review in the context of what is already known. Explain why	Yes, P-4

SECTION	ITEM	PRISMA-ScR CHECKLIST ITEM	REPORTED ON PAGE #
		the review questions/objectives lend themselves to a scoping review approach.	
Objectives	4	Provide an explicit statement of the questions and objectives being addressed with reference to their key elements (e.g., population or participants, concepts, and context) or other relevant key elements used to conceptualize the review questions and/or objectives.	Yes, P-6 ; also, see Table 1
**METHODS**			
Protocol and registration	5	Indicate whether a review protocol exists; state if and where it can be accessed (e.g., a Web address); and if available, provide registration information, including the registration number.	Yes, P-11 (archived in the BRAC JPGSPH website; will be made available by the First author on reasonable request)
Eligibility criteria	6	Specify characteristics of the sources of evidence used as eligibility criteria (e.g., years considered, language, and publication status), and provide a rationale.	Yes, P-6; see Table 1 (search strategy); the literature was searched up-to-date at the time of the study i.e., during Jan 2009 – March 2021)
Information sources*	7	Describe all information sources in the search (e.g., databases with dates of coverage and contact with authors to identify additional sources), as well as the date the most recent search was executed.	Yes, P-6,7; see Table 1 (Data Sources)
Search	8	Present the full electronic search strategy for at least 1 database, including any limits used, such that it could be repeated.	See Supplementary material 1
Selection of sources of evidence†	9	State the process for selecting sources of evidence (i.e., screening and eligibility) included in the scoping review.	Yes, see Fig.1
Data charting process‡	10	Describe the methods of charting data from the included sources of evidence (e.g., calibrated forms or forms that have been tested by the team before their use, and whether data charting was done independently or in duplicate) and any processes for obtaining and confirming data from investigators.	Yes, P-8-10 (Data extraction and analysis)
Data items	11	List and define all variables for which data were sought and any assumptions and simplifications made.	Yes, P-8-10 (data extraction and analysis, also table 2)
Critical appraisal of individual sources of evidence§	12	If done, provide a rationale for conducting a critical appraisal of included sources of evidence; describe the methods used and how this information was used in any data synthesis (if appropriate).	Yes, P-5
Synthesis of results	13	Describe the methods of handling and summarizing the data that were charted.	Yes, P-8-10
**RESULTS**			

Supplement to: Islam BZ, Tune SN, Naher N, Ahmed SM. Trauma care scenarios following road traffic crashes in Bangladesh: a scoping review. *Glob Health Sci Pract*. 2023;11(2):e2200053. https://doi.org/10.9745/GHSP-D-22-00053

SECTION	ITEM	PRISMA-ScR CHECKLIST ITEM	REPORTED ON PAGE #
Selection of sources of evidence	14	Give numbers of sources of evidence screened, assessed for eligibility, and included in the review, with reasons for exclusions at each stage, ideally using a flow diagram.	See Fig. 1
Characteristics of sources of evidence	15	For each source of evidence, present characteristics for which data were charted and provide the citations.	See Table 3, P-11-41 and See ref list
Critical appraisal within sources of evidence	16	If done, present data on critical appraisal of included sources of evidence (see item 12).	Not attached due to large volume. Can be made available by the First author on reasonable request.
Results of individual sources of evidence	17	For each included source of evidence, present the relevant data that were charted that relate to the review questions and objectives.	Yes, P-11-41 (Table-3); P-41-43 (Supplementary material 3 & 4)
Synthesis of results	18	Summarize and/or present the charting results as they relate to the review questions and objectives.	Yes, P-11-16, 41-43
**DISCUSSION**			
Summary of evidence	19	Summarize the main results (including an overview of concepts, themes, and types of evidence available), link to the review questions and objectives, and consider the relevance to key groups.	Yes, P-43-46
Limitations	20	Discuss the limitations of the scoping review process.	Yes, P-47 (Limitations).
Conclusions	21	Provide a general interpretation of the results with respect to the review questions and objectives, as well as potential implications and/or next steps.	Yes, P-47 (Conclusions)
**FUNDING**			
Funding	22	Describe sources of funding for the included sources of evidence, as well as sources of funding for the scoping review. Describe the role of the funders of the scoping review.	Yes, It is mentioned in the financial disclosure system.

Supplement to: Islam BZ, Tune SN, Naher N, Ahmed SM. Trauma care scenarios following road traffic crashes in Bangladesh: a scoping review. *Glob Health Sci Pract*. 2023;11(2):e2200053. https://doi.org/10.9745/GHSP-D-22-00053

Supplement 3. Summary of the reviewed reports and documents

Title & Date	Institution/ Author	Key findings
Regional report on the status of road safety: the south-east Asia region (2009)	World Health Organization	• This survey found that approximately 2,88,768 people were killed in 2007 on the roads in 10 of the 11 countries that make up the WHO South-East Asia Region. Almost three-quarters of road traffic deaths were among vulnerable road users (pedestrians, motorcyclists and cyclists). All the participating countries reported at least one law related to the five major risk factors (speed, drink-driving, helmets, seatbelts and child restraints) at the national or sub-national level. However, these are not all comprehensive in scope. Existing laws appear to be inadequately enforced in most countries.
Road Safety in Bangladesh Ground Realities and Action Imperatives (2013)	BRAC and PPRC	• Immediate post-crash needs include first aid, transportation, emergency medical treatment, and protection. The longer-term needs can consist of long-term therapy, assistive devices, rehabilitation, psychosocial and emotional support, and economic support and employment.<br>• It is essential to develop a universally valid national access emergency number.
ADB road safety programs for roads & highways Department (2013)	International Road Assessment Programme	• A nationwide cross-sectional survey of households in Bangladesh found that injuries resulting from road crashes are the second leading cause of permanent disability in the country<br>• There are no processes or guidelines in Bangladesh to ensure the provision of emergency medical services to road crash victims at the crash site and protect "Good Samaritans" who provide roadside care to victims.<br>• Bangladesh will require an estimated additional investment of US$7.8 billion over the coming decade to achieve the Sustainable Development Goal 3.6 target of a 50 per cent reduction in national road crash fatalities.
Bangladesh: Road Safety Improvement Programs (2013)	Asian Development Bank	• Pedestrian accidents represent an overwhelming majority of fatal accidents. Even children are also at risk.<br>• A review of the available accident data has identified that trucks and buses are involved in the majority of the accidents. One-third of

		pedestrians involved in accidents are hit by a truck. Buses hit another quarter of pedestrians, followed by minibuses and motorcycles. Furthermore, accidents with overturned vehicles and head-on collisions with buses and trucks kill hundreds of road users travelling on a low-cost budget.
Road Safety in Bangladesh: State-of-the-art and actions for Sustaina ble Transport Deve lopment (2014)	Accident Research I nstitute (ARI), Bangl adesh University of Engineering & Te chnology (BUET)	• The Road Safety Action Plan identified nine prio rity sector activities for improving road safety. T hese are:<br>I. Planning, Management and Co-ordination of Road safety<br>II. Road Traffic Accident Data System<br>III. Road Safety Engineering<br>IV. Road and Traffic Legislation<br>V. Traffic Enforcement<br>VI. Driver Training and Testing<br>VII. Vehicle Safety<br>VIII. Road Safety Education and Publicity<br>IX.<br>Medical Services for Road traffic Accident Victi ms
Road Safety Problems In Bangladesh; Dimension & Consequences (2015)	Accident Research Institute (ARI), Bangladesh University Of Engineering And Technology (BUET)	• Accidents are highly clustered: nearly 50% of accidents in less than 5% of the highway network.<br>• Proposed Road safety countermeasures: Improvement of the shoulder (hard and soft shoulder, vertical drop), Removal of visual obstruction (permanent and temporary), Access control (major-minor road connections, frequent median opening), Roadside hazard and parking management (bazaar, vendors, illegal parking), Curve improvement (superelevation, widening)
Global Status Report on Road Safety (2018)	World Health Organization	• The national emergency access number has partial coverage, no trauma registry, no formal certification for pre-hospital providers and no national assessment of emergency care systems.<br>• WHO estimated road traffic fatalities were 24,954 in 2016<br>• WHO estimated rate per 100 000 population was 15.3 in 2016<br>• Partial audits or star rating required for new road infrastructure

		• Design standards for the safety of pedestrians /Cyclists are present<br>• Inspections/ star rating of existing roads are present.<br>• Investments to upgrade high-risk locations are present<br>• Policies & investment in urban public transport is present.
Delivering Road Safety in Bangladesh: Leadership Priorities and Initiatives to 2030	The World Bank	• Between 1990 and 2017, the increase in the road crash fatality rate per capita was three times higher in Bangladesh than that across the South Asia region.<br>• Road safety performance in Bangladesh is not just flawed; it is deteriorating. Estimates of annual deaths in road accidents range from 2,538 to nearly 10 times that—between 20,736 and 21,316 (WHO 2015)<br>• Currently, less than 10 per cent of all seriously injured crash victims in Bangladesh are transported by ambulance to their first point of emergency care. Ambulances are operating in a disjointed and scattered way without a clear policy<br>• The disproportionate impact of road crash mortality and morbidity on the economically productive segment of the population is likely to depress GDP growth rates.

Supplement to: Islam BZ, Tune SN, Naher N, Ahmed SM. Trauma care scenarios following road traffic crashes in Bangladesh: a scoping review. *Glob Health Sci Pract*. 2023;11(2):e2200053. https://doi.org/10.9745/GHSP-D-22-00053

Supplement 4. Role of different NGOs, Voluntary and Civil Society Organization

Institutions	Activities
Niparad Sarak Chai (NISCHA)	• *Creation of educated drivers and transport labours*: conducted this project in 40 districts and provided awareness training on road safety to 40000 professional drivers till date with the support of Asian Development Bank (ADB), Deutsche Gesellschaft für Internationale Zusammenarbeit (GIZ), Kreditanstalt für Wiederaufbau (KfW- formerly KfW Bankengruppe) and the American Centre.<br>• *Refresher training of existing drivers and transport labours*: A full day refresher training program on road safety for professional drivers to create awareness and motivate them to drive safely. This is an ongoing project of NISCHA.<br>• *School programme*: Till date, over 30,000 students across more than 600 schools and colleges, since the year 2015, have been given road safety awareness training and workshops across Bangladesh.
Traumalink	• *A volunteer-based emergency response system* to reduce the number of deaths and injuries on the highways. Their service model utilises an emergency hotline number, a 24/7 call centre, and volunteer first responders recruited from the local community who are trained in basic trauma first aid and provided with necessary medical equipment like stretchers and bandages and dispatched to crash scenes by SMS messaging to provide medical treatment during the golden hour.<br>• After starting their function in 2013, they are currently operating on a total of 135 km: 65 km on the Dhaka-Chittagong Highway in Munshiganj and Cumilla, 40 km on the Dhaka-Aricha Highway in Dhaka and Manikganj, and 30 km on the Dhaka-Mymensingh Highway in Gazipur, working with 545 volunteers who have already responded to nearly 1300 emergency calls and treated over 2700 injured patients.<br>• The organisation provided emergency first aid training to over 1200 people, including our volunteers, university students, and youth organisations.
Criticalink	• A non-profit social business to save lives in Bangladesh by training volunteer First Responders in basic emergency medical skills (bleeding control, splinting of fractures, triage, burn care, CPR, etc.) and using an innovative, location-based mobile network to quickly dispatch the closest volunteers to the scene of an accident to provide help to injured citizens. So far, they have trained over 3,000 youth volunteers as first responders through a three-day training course.<br>• They operate by a mobile phone application where anyone can report an accident. The system alerts nearby first responders to provide first aid and help transport the patient to the hospital if needed. They operate mainly in Dhaka with a plan of expansion to other districts.
Centre for Rehabilitation of	• CRP uses a holistic approach to rehabilitation and the community reintegration process.

the Paralyzed (CRP)	• Their Occupational Therapy department provides therapy to RTC victims mostly.
Centre for Injury Prevention and Research, Bangladesh (CIPRB)	• Through pioneering research and innovation, it delivers evidence-based interventions throughout Bangladesh. These are designed to combat injury-based fatalities and morbidities including drowning, burns, and road traffic injuries.<br>• They have a programme for community first responders in the case of RTCs and death mapping as well as injury research.
BRAC	• In 2001, BRAC initiated its Road Safety Programme with a long-term vision to identify root causes and tackle this issue via a multi-sectoral engagement approach.<br>• The programme conceived and implemented a community road safety approach and operates a driving school that offers various training to promote Bangladesh's road safety condition.<br>• Additionally, the advocacy component of the programme facilitates awareness and action amongst policymakers and communities.
Accident Research Institute (ARI), BUET	• This is the only national institute for collecting, storing, and researching road accidents in Bangladesh.<br>• It conducts accident investigation and reconstruction, road safety audits, seminars, workshops, and training on road safety for professionals.<br>• It also collaborates with different government and non-government agencies and provides advisory services regarding road safety issues.
Jaatri Kollyan Samiti (Passenger welfare Association)	• This non-profit organisation mainly presents the summary of RTCs monthly.
